# An Integrated Epigenetic and Genetic Analysis of DNA Methyltransferase Genes (*DNMT*s) in Tumor Resistant and Susceptible Chicken Lines

**DOI:** 10.1371/journal.pone.0002672

**Published:** 2008-07-16

**Authors:** Ying Yu, Huanmin Zhang, Fei Tian, Wensheng Zhang, Hongbin Fang, Jiuzhou Song

**Affiliations:** 1 Department of Animal and Avian Sciences, University of Maryland, College Park, Maryland, United State of America; 2 Agriculture Research Service (ARS), United States Department of Agriculture (USDA), Avian Disease and Oncology Laboratory, East Lansing, Michigan, United State of America; 3 Division of Biostatistics of The University of Maryland Greenebaum Cancer Center, University of Maryland School of Medicine, Baltimore, Maryland, United State of America; University of Minnesota, United States of America

## Abstract

Both epigenetic alterations and genetic variations play essential roles in tumorigenesis. The epigenetic modification of DNA methylation is catalyzed and maintained by the DNA methyltransferases (*DNMT3a*, *DNMT3b* and *DNMT1*). DNA mutations and DNA methylation profiles of *DNMT*s themselves and their relationships with chicken neoplastic disease resistance and susceptibility are not yet defined. In the present study, we analyzed the complexity of the DNA methylation variations and DNA mutations in the first exon of three *DNMT*s genes over generations, tissues, and ages among chickens of two highly inbred White Leghorn lines, Marek's disease-resistant line 6_3_ and -susceptible line 7_2_, and six recombinant congenic strains (RCSs). Among them, tissue-specific methylation patterns of *DNMT3a* were disclosed in spleen, liver, and hypothalamus in lines 6_3_ and 7_2_. The methylation level of *DNMT3b* on four CpG sites was not significantly different among four tissues of the two lines. However, two line-specific DNA transition mutations, CpG→TpG (Chr20:10203733 and 10203778), were discovered in line 7_2_ compared to the line 6_3_ and RCSs. The methylation contents of *DNMT1* in blood cell showed significant epimutations in the first CpG site among the two inbred lines and the six RCSs (*P*<0.05). Age-specific methylation of *DNMT1* was detected in comparisons between 15 month-old and 2 month-old chickens in both lines except in spleen samples from line 7_2_. No DNA mutations were discovered on the studied regions of *DNMT1* and *DNMT3a* among the two lines and the six RCSs. Moreover, we developed a novel method that can effectively test the significance of DNA methylation patterns consisting of continuous CpG sites. Taken together, these results highlight the potential of epigenetic alterations in *DNMT1* and *DNMT3a*, as well as the DNA mutations in *DNMT3b*, as epigenetic and genetic factors to neoplastic diseases of chickens.

## Introduction

The relationship between somatic epigenetic variations and genetic mutations of germlines has been an emerging area in exploring tumorigenesis [1], [2]. Epigenetic and genetic mechanisms all contribute to the development of cancers [3], [4], [5], [6]. Single nucleotide polymorphisms (SNPs) are quite common genetic mutations in general populations, and one third of SNPs in humans have been associated with increased or decreased risks of various diseases, including cancers [7], [8], [9], [10], [11], [12]. In contrast, epigenetics, first described by Conrad Waddington six decades ago, is defined as the heritable changes in gene expression that occur without an alteration in DNA sequence [13]. Aberrant DNA methylation, one of the epigenetic modifications, is mitotically heritable. The genomes of cancer cells are often hypomethylated in repetitive elements and hypermethylated in the promoter and/or the first exon region of tumor suppressor genes compared to their normal counter-parts [14], [15].

Three mainly active DNA methyltransferase genes, including *DNMT*1, *DNMT3a* and *DNMT3b*, are involved in the epigenetic control of DNA methylation processes at the cytosine of CpG dinucleotides [16], [17]. *DNMT1* is the most abundant DNA methyltransferase in mammalian cells, and is considered to be the key maintenance methyltransferase in mammals [18], [19], [20]. *DNMT3a* and *DNMT3b* function as *de novo* methyltransferases and together are responsible for methylation pattern acquisition during gametogenesis, embryogenesis, and somatic tissue development as well as maintaining the silence of transposable elements and enhancing the stability of genome [21], [22], [23], [24]. The DNA methyltransferases are not limited to catalyzing DNA methylation, but also involved in the regulation of gene expression. For example, *DNMT3l*, the regulator of *DNMT3a* and *DNMT3b*, was identified to have lost DNA methylation level in cervival cancer patients [25]; DNA mutations in *DNMT3b* are of importance in cancer progression [26], [27] and ICF syndrome (immunodeficiency, centromere instability and facial anomalies, an autosomal recessive disease) in humans [28]. However, the epigenetic DNA methylation alterations and DNA mutations of *DNMT*s themselves and their association with the resistance or susceptibility of neoplastic disease are still poorly understood.

Neoplastic diseases, defined as any malignant growth or tumor resulting from abnormal or uncontrolled cell division, are serious problems in animal and human health [29], [30]. The tumors may spread to other parts of the body through the lymphatic system or the blood stream. Being a neoplastic disease, Marek's disease (MD) is not only a natural model for lymphomas overexpressing Hodgkin's disease, but a serious concern to the poultry industry as a cause of economical losses due to the cost of routine vaccination against MD [31], [32], [33], [34]. In order to explore the epigenetic and genetic background and develop better strategies in preventing the outbreak of neoplastic diseases in chickens, we initiated explorations to determine if the DNA methylation status and the single nucleotide polymorphisms (SNPs) of chicken DNA methyltransferase genes are related to susceptibility or resistance of neoplastic diseases. We tested this hypothesis in two unique chicken inbred lines (6_3_ and 7_2_) through pyrosequencing, sequencing, and quantitative RT-PCR analyses. The highly inbred line 6_3_, developed and maintained at the Avian Disease and Oncology Laboratory (ADOL), is highly resistant to Marek's disease (MD) tumors induced by the MD virus (MDV), whereas the highly inbred line 7_2_ is highly susceptible to MD tumors [35]. Moreover, based on the two inbred lines, 19 recombinant congenic strains (RCS) have been developed. These are the only RCSs in livestock species. This unique chicken model system provides a way to elucidate epigenetic and genetic mechanisms that may influence the susceptibility or resistance to neoplastic diseases [30], [36].

To examine the hypothesis, we first investigated tissue and age specific methylation profiles in the first exons of *DNMT1*, *DNMT3a*, and *DNMT3b* among chickens from the MD-resistant line 6_3_, MD-susceptible line 7_2_, and six RCSs by pyrosequencing technology. Pyrosequencing is a quantitative technique used to detect changes in DNA methylation patterns. This technique is advantageous for analyzing and quantifying the degree of methylation of multiple continuous CpG sites in one reaction [37]. Subsequently, DNA mutations were detected by PCR sequencing. Next, we examined the mRNA expression levels of the genes using quantitative RT-PCR. Finally, we explored a new statistical method to classify and test the methylation patterns. The relationship of epigenetic variations and genetic mutations of the *DNMT* genes between the two inbred lines was discussed. Our results suggested that the aberrant methylation profiles of *DNMT1* and *DNMT3a* as well as CpG to TpG transitions in *DNMT3b* are complex epigenetic and genetic factors that may be involved with neoplastic disease susceptibility or resistance in chickens.

## Results

### The sketch of DNA methylation analysis of *DNMTs* in the inbred lines and RCSs of chickens

**Figure 1:**
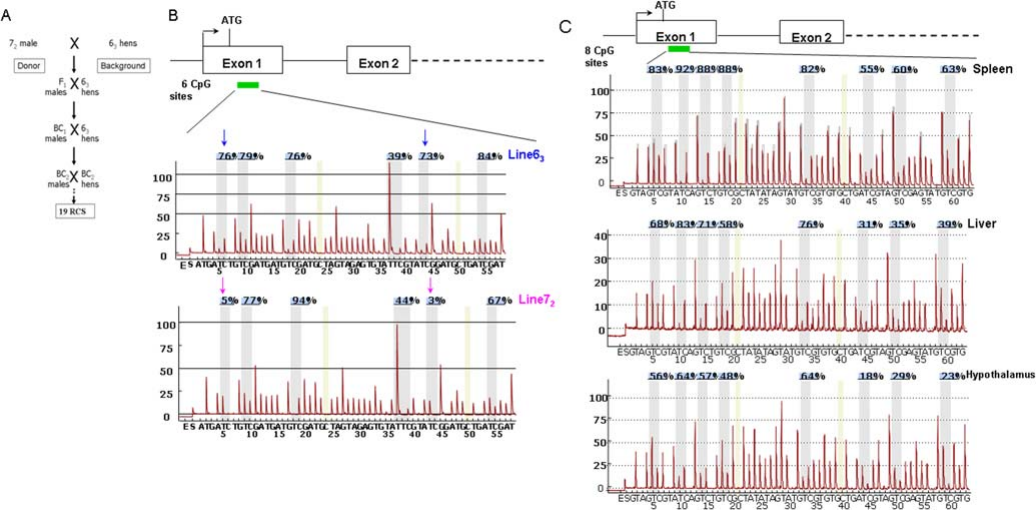
Schematic structure of developing the recombinant congenic strains (RCSs) and quantifying DNA methylation levels of *DNMT3b* and *DNMT3a* genes. A. The strategies to develop the 19 RCSs from the background line 6_3_ and donor line 7_2_; B. Upper panel, methylation analysis region on the first exon of *DNMT3b*. Position of transcription start site (black arrow), translation start code (ATG) and exons (open boxes) based on NCBI database (ID NM_001024828). Green box shows the position of the analyzed 6 CpG sites. Lower panel, DNA methylation pyrograms of six CpG sites in the first exon of *DNMT3b* in line 6_3_ and line 7_2_ using Pyro Q-CpG system. Grey areas indicate the CpG sites that were analyzed. The yellow regions indicate internal control regions for automatic assessment of bisulfite conversion (unmethylated C should be fully converted to T). Blue arrows and pink arrows show the methylation contents in CpG sites 1 and 5 in line 6_3_ and line 7_2_, respectively. C. Upper panel, methylation analysis region of 8 CpG sites on the first exon of *DNMT3a* (NCBI ID: 10HNM_001024832). Lower panel, DNA methylation pyrograms of 8 CpG sites in the first exon of *DNMT3b* in spleen, liver and hypothalamus. The percentages in each CpG site is the methylation percentage of ^m^C/(^m^C+C) on this site. ^m^C: methylated cytosine, C: unmethylated cytosine.

Previous studies have documented the distinct features of the chicken inbred lines 6_3_ and 7_2_ on neoplastic disease resistance or susceptibility as well as the genetic backgrounds of recombinant congenic strains (RCSs) [29], [36], [38], [39], [40]. Figure 1A shows that RCSs were intentionally established by crossing Marek's disease-resistant line 6_3_ and -susceptible line 7_2_. The F_1_ was then consecutively backcrossed to the background line 6_3_ twice. A series of 19 RCSs were established by sib-mating of the second backcross (BC2) chickens. Each RCS on average carries a random sample of 87.5% background line 6_3_ genome and 12.5% donor line 7_2_ genome. The RCSs and their parental lines are a valuable resource for genetic and epigenetic analysis of neoplastic disease resistance and susceptibility.

In this study, we firstly sought to explore the epigenetic variations of DNA methylation for the three DNA methyltransferase genes (*DNMT3a*, *DNMT3b* and *DNMT1*) among the two parental lines and six RCSs through pyrosequencing technology. We designed PCR primers and sequencing primers for methylation assays on the first exon of each *DNMT* gene as shown in Table 1 and Figure 1B and 1C. Pyrosequencing methylation analysis is based on using bisulfite converted DNA and PCR to quantitatively evaluate the methylated cytosine (^m^C) from unmethylated cytosine (C), and to quantify the methylation ratio by ^m^C/(^m^C+C) at each CpG site. Pyrograms via pyrosequencing technology exhibit quantitative methylation level in each CpG site. As illustrated in Figure 1B, *DNMT3b* has different methylation profiles on the first exon in two highly inbred lines in blood cell. The methylation contents of *DNMT3b* at CpG sites 1 and 5 is seen to be much higher in line 6_3_ (76% and 73%, respectively) than in line 7_2_ (5% and 3%) in blood cell, whereas other 4 CpG sites show no obvious differences between the two lines. From Figure 1C, it is clear that *DNMT3a* shows different methylation profiles in spleen, liver, and hypothalamus, *i.e.*, the average methylation contents of *DNMT3a* at 8 CpG sites were much higher in spleen (76.4%±14.6%) compared to that in liver (57.6%±20.1%) and in hypothalamus (44.75%±18.9%).

**Table 1:** PCR and pyrosequencing primers and assays for *DNMT* genes.

Gene^A^	Assay CpG sites^B^	Primers	Sequence^C^
*DNMT3b*	6: 10203733	Forward	5′-GAGGGTTTTTTGGTTGGTTAAGT-3′
	10203737	Reverse	5′-GGGACACCGCTGATCGTTTA CCTCCAACAACAAAACAACAATAT-3′
	10203747	Sequencing	5′-GTTATGAAAAAGGAGAAGAG-3′
	10203775	Assay sequence	5′-TTA**Y**GGT**Y**GG GATGAGG**Y**GG ATTGTAGGGT AGAGTTGATTTTTTT **Y**GA**Y**G
	10203778		GGGATTGTAT **Y**GATTTTATT-3′
	10203790		
*DNMT3a*	8: 107432308	Forward	5′-GGTTYGTCGTYGGTTGATTTG-3′
	107432311	Reverse	5′-GGGACACCGCTGATCGTTTAAACCCCCCTACCTCACAACAAC-3′
	107432314	Sequencing	5′-TTGATTTGGATGTGTTTT-3′
	107432317	Assay sequence	5′-GT**Y**GA**Y**GG**Y**GT**Y**GGTTATTATAGGTTTAGG**Y**GTGGTGGTTA**Y**GTTGGG**Y**GA
	107432336		GGTAGGGT **Y**GGTGGGTTG -3′
	107432347		
	107432354		
	107432365		
*DNMT1*	4: 49757939	Forward	5′-TGGGAAGAGGAAGGGGATAT-3′
	49757957	Reverse	5′-GGGACACCGCTGATCGTTTA TCCCCAATAAAATCTCCTACCA-3′
	49757946	Sequencing	5′-GGAAGAGGAAGGGGATA-3′
	49757980	Assay sequence	5′-T**Y**GATTTTTTGGAAAATA**Y**GTTTAGGATGGTGGGTTGT**Y**G GT**Y**GGAGTTG-3′
		Universal	5′-/Biotin labeled/GGGACACCGCTGATCGTTTA-3′

^A^: Gene name. ^B^: *DNMT3b*, based on the UCSC DNA sequence (May 2006, Chr20) that BLAT from *DNMT3b* cDNA sequence (NCBI: NM_001024828), it is in the exon1 region of *DNMT3b*. *DNMT3a*, based on the UCSC DNA sequence (May 2006, Chr3) that BLAT from *DNMT3a* cDNA sequence (NCBI: NM_001024832), it is in the exon1 region of *DNMT3a*. *DNMT1*, based on the UCSC DNA sequence (May 2006, Chr-Un-random) that BLAT from predicted *DNMT1* DNA sequence (NCBI: NM_001475597). ^C^: Y and R stand for C/T and G/A, respectively. Bold Y is the CpG sites assayed in each gene.

### DNA methylation profiles of *DNMT3b* in different lines, tissues and ages

**Figure 2:**
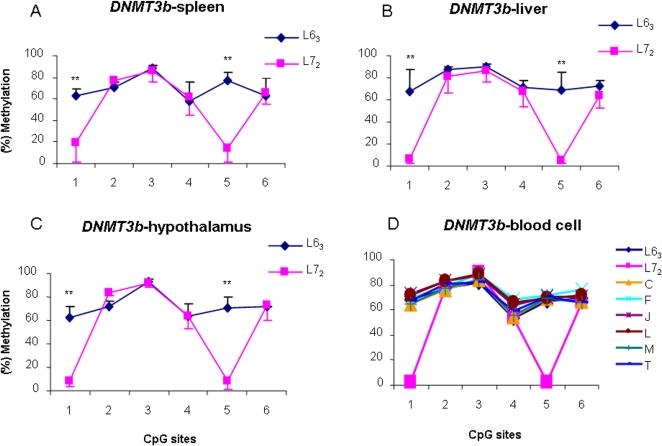
DNA methylation variations of *DNMT3b* in spleen (A), liver (B) and hypothalamus (C) in line 6_3_ and 7_2_, and in blood cell (D) in the two parental lines and the six recombinant congenic lines. L6_3_: lines 6_3_; L7_2_: lines 7_2_. C, F, J, L, M and T: six recombinant congenic strains. *n* = 5 for each line and RCS.

**Figure 3:**
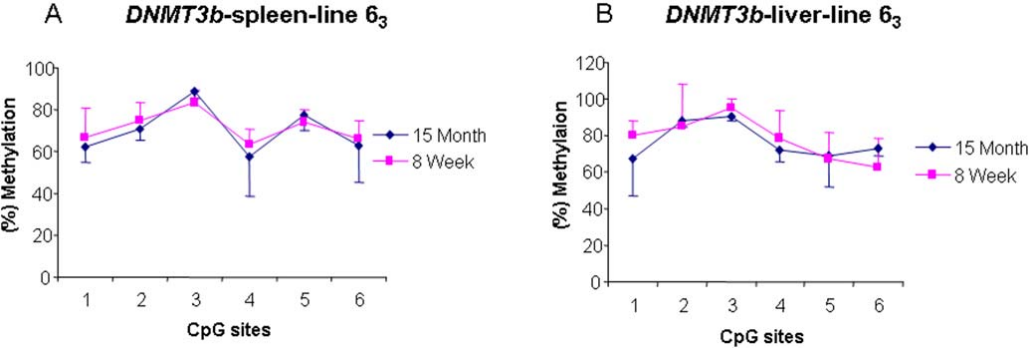
DNA methylation profiles of *DNMT3b* in spleen (A) and liver (B) at 15 months and 8 weeks old in the line 6_3_. *n* = 5 for each line.

To further check the variations of DNA methylation patterns of the three chicken *DNMTs*, we examined DNA methylation levels of *DNMT3b* in four tissues at two ages of the two lines. Figure 2A, 2B, and 2C exhibited the methylation profiles of the *DNMT3b* in the spleen, liver and hypothalamus between lines 6_3_ and 7_2_ at 15 months of age. We found that the methylation levels of *DNMT3b* at CpG sites 1 and 5 were significantly higher in line 6_3_ than in line 7_2_ (*P*<0.01). We thereafter examined the methylation status of *DNMT3b* in spleen and liver in line 6_3_ chickens of 2 months of age. As shown in Figure 3A and 3B, the methylation level of *DNMT3b* gene in spleen and liver shows a similar pattern in line 6_3_ chickens of 2 and 15 months of ages (*P*>0.05). The results implied that the DNA methylation of *DNMT3b* does not have age specificity in line 6_3_.

Having observed distinct methylation patterns of *DNMT3b* in the parental lines 6_3_ and 7_2_, to check the epigenetic inheritance or epimutations of DNA methylation of the gene, we measured the methylation status of the *DMNT3b* gene in blood cell from the two parental lines and six RCSs, C, F, J, L, M and T. As shown in Figure 2D, we found that, in the eight chicken strains, the methylation patterns can easily be categorized into two groups, one of which is extremely similar to each other among 6 RCSs, which also closely resembles the background line 6_3_ in contrast to the donor line 7_2_. The results imply that the *DNMT3b* shows transgenerational epigenetic inheritance from background line 6_3_ to six RCSs through meiosis for more than thirteen generations.

### Line-specific SNPs and discrepant mRNA expressions of *DNMT3b*

**Figure 4:**
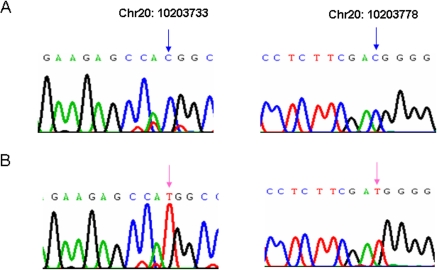
Two CpG to TpG transitions in the first exon of *DNMT3b* by DNA sequencing. A. Blue arrows show the cytosines in CpG sites 1 (Chr20: 10203733) and 5 (Chr20: 10203778) of *DNMT3b* in the line 6_3_, RCSs (*n* = 3 for each strain) and red jungle fowl (*n* = 1). B. Pink arrows show the two CpG to TpG transitions in the line 7_2_. *n* = 3.

The extremely low methylation levels in the CpG sites 1 and 5 of *DNMT3b*, compared to other 4 CpG sites, suggested that this difference could be attributed to single nucleotide polymorphisms (SNPs) between the two inbred lines. Using DNA sequencing thereafter, two CpG to TpG transitions located in the same CpG sites 1 and 5 for DNA methylation analysis, were found in line 7_2_ (Figure 4B) but not in line 6_3_, the RCSs, or a wild-type chicken, a red jungle fowl (Figure 4A). Taking all this into consideration, the two line-specific SNPs of *DNMT3b* appear to be possible genetic factors that are involved in MD tumor susceptibility in the line 7_2_.

**Figure 5:**
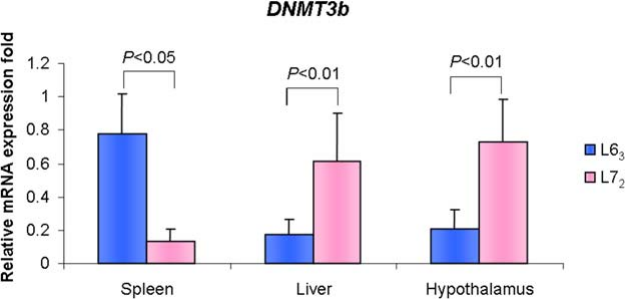
mRNA expression differences of *DNMT3b* in spleen, liver and hypothalamus between line 6_3_ and line 7_2_ at 15 months-old with quantitative RT-PCR. Two replicates for each reaction. *n* = 5 for each line.

SNPs located in promoter/exon1 region of a gene may negatively or positively regulate the expression level of the gene. To ascertain the relationships, we then measured the level of *DNMT3b* mRNA in spleen, liver, and hypothalamus of 15 month-old chickens from the two parental lines via real-time quantitative RT-PCR. Unexpectedly, as shown in Figure 5, the mRNA level of *DNMT3b* in spleen was found to be significantly higher in line 6_3_ than in line 7_2_ (*P*<0.05), whereas it was significantly lower in liver and hypothalamus of line 6_3_ than line 7_2_ (*P*<0.01). The results suggested that *DNMT3b* may be involved in complicated regulation and/or expression mechanisms.

### Tissue-specific methylation variations of *DNMT3a* in the line 7_2_ and line 6_3_

**Figure 6:**
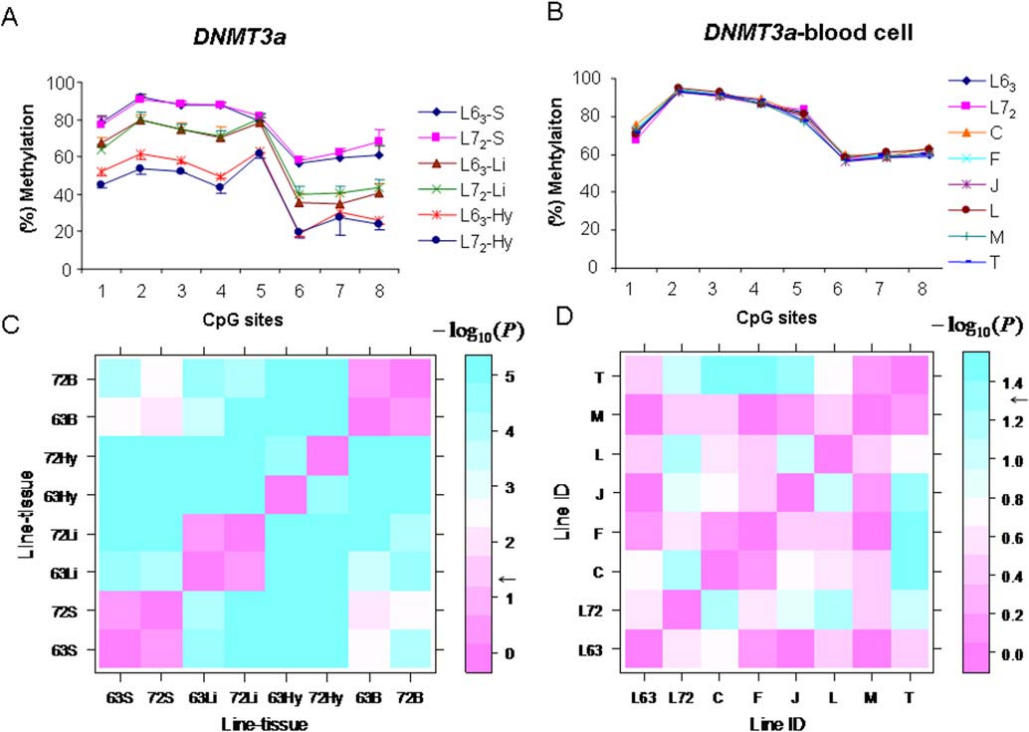
Tissue-specific DNA methylation variations and patterns analysis of *DNMT3a* in spleen, liver and hypothalamus (A) in line 6_3_ and line 7_2_, and in blood cell (B) in two lines and six RCSs, C, F, J, L, M and T. L6_3_: line 6_3_; L7_2_: line 7_2_. S, spleen; Li: liver; Hy: hypothalamus. *n* = 5 for each line and RCS. C. *P* values matrix among lines and tissues using exact F test for DNA methylation patterns of *DNMT3a*. 63: line 6_3_; 72: line 7_2_. *n* = 5 for each. D. *P* values matrix for DNA methylation patterns of *DNMT3a* among two parental lines 6_3_ and 7_2_, as well as six RCSs. *n* = 5 for each. Color bar shows the extent of significance level (*P* values with −*log*_10_(*P*). *e.g.*, −*log*_10_(0.05) = 1.3; −*log*_10_(0.01) = 2). Black arrows show *P* = 0.05.

*DNMT3a* is another *de novo* DNA methyltransferase like *DNMT3b*. To examine the methylation profiles of *DNMT3a*, we conducted DNA methylation analysis of *DNMT3a* in tissues of chickens from the two age groups of the lines. As shown in Figures 6A, we found that the eight continuous CpG sites of *DNMT3a* displayed tissue-specific methylation patterns in both lines at 15 months of age, meaning that the highest level of DNA methylation was found in spleen (75.2%±2.5% in line 6_3_ and 76.6%±2.5% in line 7_2_) among the three tissues, followed by liver (line 6_3_ = 60.1%±4.0%, line 7_2_ = 61.8%±3.4%), and the lowest level was found in the hypothalamus (44.9%±1.7% in line 6_3_, 40.8%±3.2% in line 7_2_). Quantitative RT-PCR demonstrated that the mRNA expression of *DNMT3a* was significantly higher in spleen than in liver in the line 6_3_ (*n* = 5, Figure S1A).

**Figure 7:**
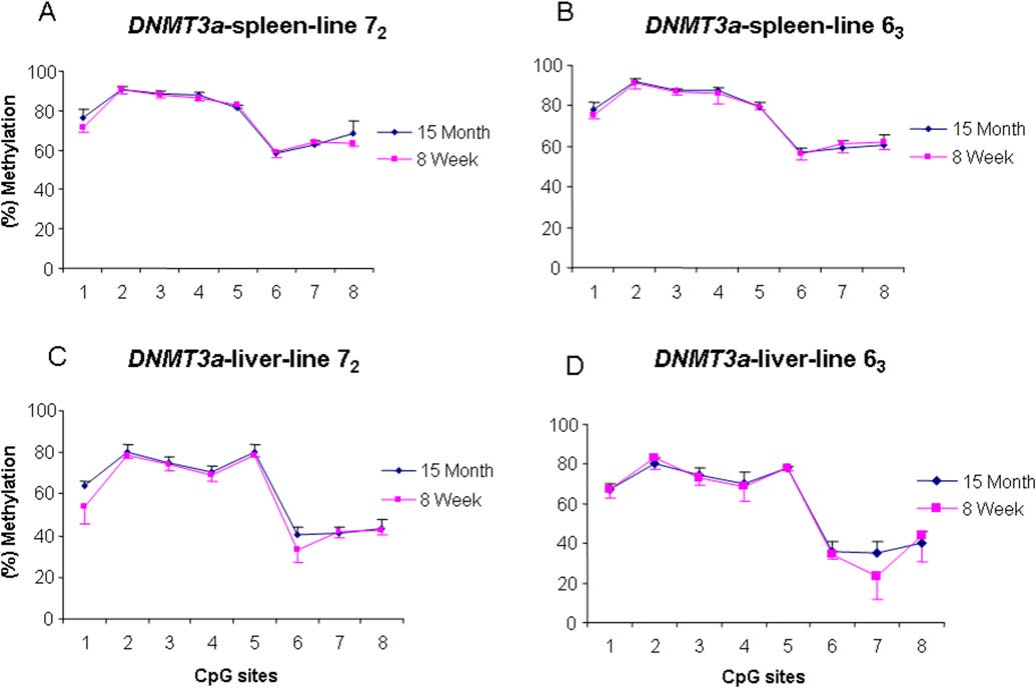
DNA methylation profiles of *DNMT3a* in spleen (A, B) and liver (C, D) at 15 months and 8 weeks old in line 6_3_ and line 7_2_. *n* = 5 for each line.

Interestingly, we noticed that the methylation levels of *DNMT3a* were very similar to each other in the two lines, but only one tissue, the hypothalamus, among three tissues, showed significantly higher methylation level in line 6_3_ than in line 7_2_ (*P*<0.01) at the first four CpG sites (Figure 6A). Like *DNMT3b*, the methylation level of *DNMT3a* gene also showed a similar pattern across the two ages (*P*>0.05), 2 month and 15 month old chickens , in spleen (Figure 7A and 7B) and liver (Figure 7C and 7D) of both lines. In addition, as shown in Figure 6B, DNA methylation patterns of *DNMT3a* in the two parental lines and six RCSs are almost the same as that in the blood cell at 12 months of age. These results indicate that, compared to *DNMT3b*, *DNMT3a* is a tissue-specific *de novo* DNA methyltransferase, and its methylation patterns and expression levels varied among the tissues.

### Age-specific DNA methylation variations of *DNMT1*

**Figure 8:**
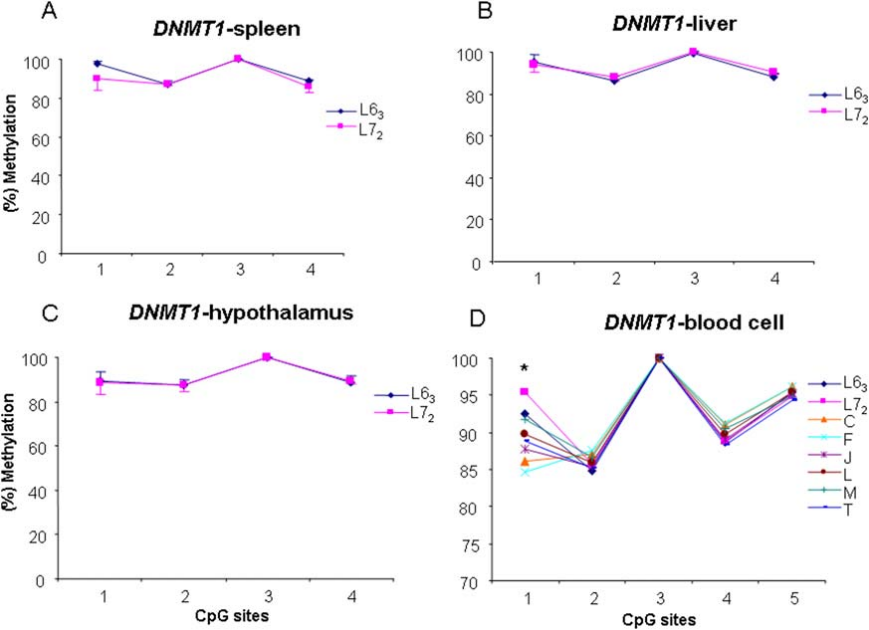
DNA methylation variations of *DNMT1* in spleen (A), liver (B) and hypothalamus (C) in the lines 6_3_ and 7_2_, and in blood cell (D) in the two parental lines and six recombinant congenic lines. L6_3_: lines 6_3_; L7_2_: lines 7_2_. C, F, J, L, M and T: six recombinant congenic strains. * *P*<0.05. *n* = 5 for each line and RCS.

**Figure 9:**
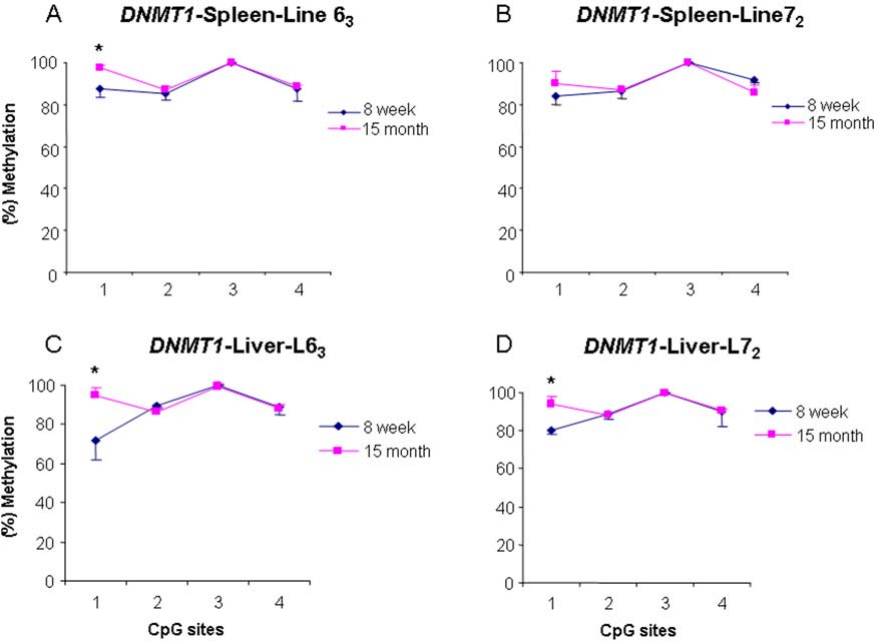
DNA methylation variations of *DNMT1* in spleen (A, B) and liver (C, D) at 15 months and 8 weeks old in line 6_3_ and line 7_2_. * *P*<0.05. *n* = 5 for each line.

To maintain methylation patterns in daughter cells during DNA replication, the unmethylated daughter CpG site opposite to a methylated parental CpG site must be methylated by the maintenance methyltransferase, *DNMT1*. It is important, therefore, to investigate the methylation status of *DNMT1* and compare it with the *de novo* methyltransferases in the unique chicken population. We quantitatively measured DNA methylation levels of four CpG sites in the exon1 region of *DNMT1* in four tissues at three age stages. Our results indicated that *DNMT1* had a similar methylation pattern in the spleen, liver, and hypothalamus (Figure 8A, 8B and 8C) between the two inbred lines at 15 months of age, whereas the CpG site 1 in blood cell showed a significant epimutation among the two parental lines and the six RCSs (*P*<0.05) (Figure 8D) at 12 months of age. Interestingly, comparing the methylation levels of *DNMT1* between the two ages, we found that the methylation level of the CpG site 1 at two months of age was lower than that at 15 months of age in spleen (*P*<0.05) (Figure 9A) and liver (Figure 9C and 9D) for both the parental lines except in spleen of the line 7_2_ (Figure 9B). The results indicated that *DNMT1* is an epimutation gene with age-specific methylation patterns in the chickens.

### DNA methylation patterns analysis of *DNMTs* via principal component test

DNA methylation level is frequently aberrant on different CpG sites within one CpG island. Thus, analyzing only the average of methylation levels in continuous CpG sites may blur the major effect of the single CpG site. On the other hand, point-wise comparison can only be used to analyze the methylation difference of the same CpG site between different samples. To explore which CpG site in a continuous CpG sites region (CpG islands) is mainly responsible for the fluctuation of the methylation level and classification of methylation patterns, we developed a new method to quantitatively evaluate the methylation patterns of *DNMTs* via a nonparametric analysis method that transforms principle component analysis (PCA) as an exact F test.

**Figure 10:**
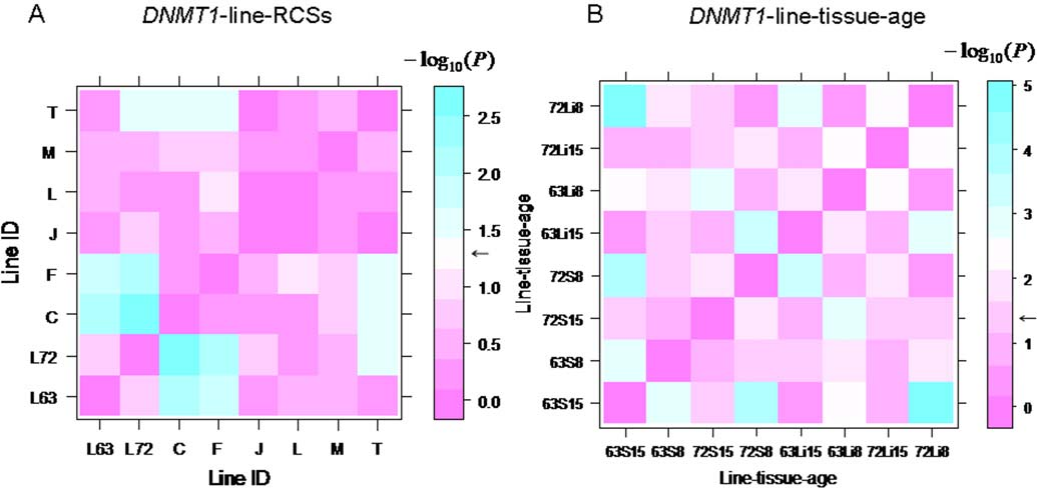
Exact F test for DNA methylation patterns of *DNMT1*. A. *P* values matrix among the two parental lines 6_3_ and 7_2_, as well as six RCSs, C, F, J, L, M and T. *n* = 5 for each. B. *P* values matrix among the lines, tissues on ages. 63: line 6_3_; 72: line 7_2_; S: spleen; Li: liver. 15: 15 months old; 8: 8 weeks old. *n* = 5 for each. Color bar shows the significance level (*P* values with −*log*_10_(*P*). *e.g.*, −*log*_10_(0.05) = 1.3; *log*_10_(0.01) = 2). Black arrows show *P* = 0.05.

Through the exact F test, we can get the probability level of profile dissimilarity between any two groups. The *P* value matrix constructed from multiple profile comparison is plotted in Figure 6C. Pink between the groups means no significant differences (*P*>0.05), while the color gradient from pink to blue indicates the extent of significant differences (*P<0.05*). Consequently, it is easy to identify the significant tissue-specific differences of methylation patterns of *DNMT3a* among the spleen, liver, hypothalamus, and blood cell (*P*<0.001). Figure 6C shows a huge difference of methylation patterns in hypothalamus between the line 6_3_ and line 7_2_ (*P*<0.001), while no major differences in spleen, liver, or blood cell between the two lines were detected (*P*>0.05). From Figure 6D, the methylation patterns of *DNMT3a* among the two parental lines and six RCSs were not significantly different in blood cell (Figure 6B, *P*>0.05). Similarly for *DNMT1*, the differences of methylation patterns among the parental lines and RCSs in blood cell as well as in spleen and liver at two age stages were also analyzed as shown in Figure 10. From the Figure 10A, we found that RCS C and F in blood cell showed considerable discrepancy with the two parental lines in *DNMT1* methylation patterns (*P*<0.05), whereas the other four RCSs showed no difference from either of the parental lines (*P*>0.05). Age-specific methylation pattern differences of *DNMT1* at 15 months and two months of age was clearly shown in Figure 10B (*P*<0.05). Based on these results, we conclude that the description of differences and analysis of DNA methylation patterns including continuous CpG sites can be identified by this newly developed method.

## Discussion

To date, the accumulating evidence of human cancers have been shown that the increased cytosine methylation levels at CpG dinucleotides of cancer suppressor genes on promoter regions and/or exon 1 or decreased methylation levels of repeat elements throughout the whole genome are highly associated with tumorigenesis [41]. In our previous report, we found the hypermethylation pattern of endogenous virus that may be relevant for resistance against tumors in chickens [31], which encouraged us to trace and analyze the epigenetic status and genetic variations of the gene family controlling DNA methylation in the unique tumor-resistant or -susceptible chicken populations. In this study, we underlined the complexity of epigenetic variations of DNA methylation and genetic mutations for three DNA methyltransferase genes (*DNMTs*) in two highly inbred chicken lines (6_3_ and 7_2_) and six recombinant congenic strains (RCSs) derived from the two inbred lines. To our knowledge, this study is the first to explore the DNA methylation variation profiles of *DNMTs* in tumor resistant or susceptible chickens. Due to lack of knowledge about the promoter locations of these genes in animals, the pilot assessment of DNA methylation was done on the first exon of these genes. Some of these variations may help us uncover the trigger of the susceptibility or resistance to neoplastic disease in chickens.

We found that the three *DNMTs* have unique epigenetic and genetic patterns in the chicken population. Among them, *DNMT1* and *DNMT3a* showed epimutations of DNA methylation on the first exon, *i.e.*, tissue-specific methylation of *DNMT3a* and age-specific methylation of *DNMT1*. Interestingly, the age-specific style shown by DNA methylation of *DNMT1* may contribute to some forms of amyloidosis that develops with aging [42]. No DNA mutations of these two genes indicated that the regulation of related gene expression should be manipulated by epimutations of these genes. In addition, the mRNA expression data in the spleen and liver showed that high CpG methylation levels in the first exon of *DNMT3a* coincided with high transcription levels in spleen, and low methylation level coincided with low expression level in liver in line 6_3_ birds (Figure S1A). Moreover, the similar CpG methylation patterns of *DNMT1* in liver were also consistent with similar transcription levels (Figure S1B) in both lines 6_3_ and line 7_2_. The results were in good agreement with reported methylation studies, that is, the methylation level on the exons of MHC (Major Histocompatibility Complex) genes were positively related to the expression levels [43], [44]. Our unpublished data indicated that the tumor numbers induced by MDV are different among tissues in line 6_3_ and line 7_2_. Thus it is possible that the tissue-specific epimutations of *DNMT3a* is associated with the tumor number variations in different tissues.

On the contrary, CpG sites 2, 3, 4 and 6 in the first exon of *DNMT3b* (Fgiure 1) did not show statistical difference among the lines in tissues of varied age groups (Figure 2 and 3). Compared to resistant line 6_3_, the six RCSs and a wild type chicken (red jungle fowl) (Figure 5A), the “methylation differences” at CpG sites 1 and 5 (Figure 2) of *DNMT3b*, hypermethylation in line 6_3_ and unmethylation in line 7_2_, are actually due to the CpG to TpG transitions in MD-susceptible line 7_2_ (Figure 5B). A further analysis disclosed that the two transitions are synonymous mutations, but the two translation start codes (ATG) were created through the two mutations (Figure S2B). Because of the mutations, a new transcriptional binding site, PAX5 (B-cell specific activator protein), is discovered at the second mutation region in line 7_2_, which replaced the original binding site, NUDR (Nuclear DEAF-1-related protein), on the same site in line 6_3_ (Figure S2A and S2B). Therefore, we postulate that this crucial replacement might be related to susceptibility of neoplastic disease. It is reported that *DNMT3b* is an alternative splicing gene in mammals [45], [46]. Further investigations are needed to confirm whether or not there are harmonious combinations between the splicing structures, methylation status, and the two SNPs in chickens, as well as the genetic effect on chicken MD susceptibility or resistance. For the discrepant mRNA expression levels of *DNMT3b* among different tissues, that is, higher expression in line 6_3_ than in line 7_2_ in spleen, but lower expression in line 6_3_ than line 7_2_ in liver and hypothalamus, further investigations will follow to determine the effects of the DNA methylation variation on the promoter region of *DNMT3b* among different tissues and to explore any network mechanisms that may be involved.

Most importantly, the methylation patterns of the genes clearly exhibited an epigenetic inheritance of interest. To date, only a few genes have been shown to display epigenetic inheritance in mammals, such as *axin-fused* (*Axin^Fu^*) [47] and yellow agouti (*A^vy^*) [48]. This observation supports that methylation patterns could be viewed as epigenotypes referring to mitotically heritable patterns of DNA methylation at CpG sites [49], [50]. The two highly inbred parental lines, 6_3_ and 7_2_, and the RCSs constitute unique resources for exploration of the nature of *DNMT1*, *DNMT3a*, and *DNMT3b*, which allow us to quantitatively measure methylation alterations of the three *DNMTs* in order to monitor whether epigenetic inheritance or epimutations of parental methylation patterns are passed on to subsequent generations. From the detailed methylation patterns of the parental lines and the RCSs plotted in Figures 2D (*DNMT3b*), 6B (*DNMT3a*), and 8B (*DNMT1*) we recognized that the methylation pattern of *DNMT3a* and *DNMT3b* in blood cell were inherited from the background line 6_3_, whereas the methylation of *DNMT1* in blood cell showed epimutaitons at the first CpG site between the six RCSs and their two parental lines. The plasticity of methylation patterns in *DNMT1* might have offered reasonable explanation of the variability of resistance to MD among a series of 19 recombinant congenic strains (unpublished data). It is worth noting that after 13 generations of sib-mating, *DNMT3a* and *DNMT3b* in the RCSs still kept the methylation pattern of the background line 6_3_, which resulted from epigenetic transgenerational inheritance. Although the epigenotype in the epigenome is far less stable than genotype of the DNA genome, the epigenetic inheritance of *DNMT3a* and *DNMT3b* and epimutations of *DNMT1* in blood cell gives us an important clue on how to combine the epigenetic and genetic information to prevent neoplastic disease in chickens.

In our quantitative methylation analysis, DNA methylation patterns are continuous measurements of CpG sites. Traditional analysis methods based on normal distribution, such as t-test and ANOVA, are thus not suitable for the data characteristics. For pattern identification, when considering the most widely used methods, such as k-means, self-organizing map (SOM) and hierarchical clustering analysis, it has been argued recently that distance based methods generate local solutions that are not necessarily meaningful. Furthermore, identifying *a priori* the number of clusters remains, in general, an open problem. Therefore, determining the right pattern is a critical issue in epigenomic analysis. However, comparing methylation patterns between groups will allow us to test the influence of DNA methylation patterns on MD risk. In this study, we adopted a new method of analysis, known as principal component test, to classify DNA methylation patterns and validated it's feasibility in computational epigenetics. In principle, the PCA is a multivariate technique, which examines the relationships among the CpG sites by producing eigenvalue and principal component scores with variance, and then summarizes the CpG data in reduced dimensions. To assess a pattern-wise comparison, an exact F test was finally carried out to get a *P* value from the PCA transformation. This method can effectively classify the DNA methylation patterns in a continuous CpG sites region as illustrated in Figures 6, 10, S3, S4 and S5.

In summary, we characterized the unique epigenetic profiles and DNA mutations of three DNA methytransferase genes in chickens. Tissue-specific methylation pattern of *DNMT3a*, age-specific methylation pattern of *DNMT1*, and CpG to TpG transitions in *DNMT3b* might be associated with susceptibility of MD, which suggests new possibilities for etiological study, MD control, and genetic breeding of MD resistant chickens using these epigenetic and genetic factors.

## Materials and Methods

### Experimental Animals and Samples

Lines 6_3_ and 7_2_ White Leghorn chickens were initially selected at the Avian Disease and Oncology Laboratory in 1939 for tumor resistance or susceptibility induced by Marek's disease virus (MDV) and avian leukosis virus. Nineteen recombinant congenic strains (RCS) were developed using line 6_3_ as the background line and line 7_2_ as the donor line (Figure 1). Samples were collected from the two highly inbred chicken lines as well as six RCSs, M, T, F, J, C and L, which showed higher to lower MD tumor incidence difference from unvaccinated and vaccinated treatment results (our unpublished data). The line 6_3_ is resistant to MD whereas the line 7_2_ is susceptible. Heparinized blood samples were collected from each of the chickens prior to euthanasia. Five blood samples were collected from females of each parental line and each RCS at 2, 12 and 15 months of age and stored at −20°C until analyses. Tissue samples of line 6_3_ and line 7_2_ at 2 and 15 months of age were obtained from three organs: liver, spleen and hypothalamus. Tissue samples were frozen in liquid nitrogen immediately at sampling and stored at −80°C until analyses. All procedures followed standard animal care and use guidelines.

### DNA extraction and bisulfite treatment

DNA was extracted from 20 µl red blood cells or 3 mm^3^ tissue samples using the phenol-chloroform method. DNA was precipitated in ethanol, pelleted and redissolved in TE (pH 8.0), and DNA concentration was measured by a spectrophotometer (Bio-Rad). Sodium bisulfite conversion of each sample of genomic DNA (1 µg) was performed using EZ DNA Methylation Golden Kit as described in the manufacturer's instructions (ZYMO Research). Bisulfite converted DNA was eluted in 20 µl elution buffer (ZYMO Research).

### PCR and sequencing primers used in pyrosequencing assay

PCR assays were designed to amplify multiple CpG dinucleotides sites in the first exon of *DNMT1*, *DNMT3a* and *DNMT3b*. The principle of primer design is that the primers do not cover any CpG sites as far as possible. As shown in Table 1, forward and reverse primers of PCR assays and sequencing primer of pyrosequencing methylation assays were designed using PSQ Assay Design software (Biotage, Sweden). Based on the BLAT results from UCSC Genome Browser and the requirement of pyrosequencing techniques (PyroMarK ID, Biotag, Sweden), one 6-CpG-site region of the *DNMT3b* on chicken chromosome 20, 8-CpG-site region of the *DNMT3a* on chromosome 3 and 4-CpG-site region of the *DNMT1* on one of microchromosome were analyzed (Table 1, Figure 1, Figure S6A, S6B and S6C). A biotin labeled universal primer (5′-GGGACACCGCTGATCGTTTA-3′) was used in the PCR assays [51]. The 5′ end of each reverse primer was tailed with the sequence as the universal primer (Table 1) [51].

### Hot start PCR amplification

The hot start PCR was carried out in a 30 µl solution for *DNMTs*: 1.5 µl diluted bisulfite treated DNA (1∶5 dilution), 1×PCR buffer, 0.2 mM dNTPs, 0.5 µM forward primer, 0.05 µM reverse primer with universal tail, 0.45 µM biotin labeled universal primer, and 0.75 U Qiagen's Hotstar *Taq* DNA polymerase (Qiagen Inc.). PCR cycling conditions were 95°C for 15 min, followed by 50 cycles of 94°C for 30 sec, 60°C for 45 sec, and 72°C for 45 sec, and a final incubation at 72°C for 10 min. PCR product quality verification was carried out on 1.5% agarose gels with ethidium bromide.

### Pyrosequencing methylation assays

Based on the concentration of the hot start PCR product, 10∼25 µl PCR product was used for each pyrosequencing reaction. Pyrosequencing methylation analysis was carried out using the Pyro Q-CpG system (PyroMark ID, Biotage, Sweden) according to manufacture's protocol. In brief, the biotin labeled PCR products were bound to Streptavidin coated Sepharose beads (GE Healthcare Bio-sciences AB, Sweden). The Sepharose beads containing the immobilized PCR product were purified in 70% ethanol for 5 sec, denatured in Denature buffer (Biotage) for 5 sec, and washed with washing buffer (Biotage) for 10 sec using the Pyrosequencing Vacuum Prep Tool (Biotage). Then, 0.5 µM sequence primer was annealed to the purified single-stranded PCR product in annealing buffer (Biotage) and pyrosequencing was carried out using the Pyro Q-CpG system. The level of methylation was expressed for each cytosine locus on CpG sites as the percentage of ^m^C/(^m^C+C). Non-CpG cytosine residues were used as internal controls to verify bisulfite conversion.

### PCR and DNA sequencing

The methylation assayed region of *DNMT3b* was amplified by PCR from the whole genome for each sample. The forward and reverse PCR primers are 5′-GGCAGCCATGAAAAAGGAGA-3′ and 5′-GGCAGCAGTGTCCTTAGTGG-3′, respectively. PCR cycling conditions were 95°C for 15 min, followed by 30 cycles of 94°C for 30 sec, 62°C for 45 sec, and 72°C for 20 sec, and a final incubation at 72°C for 10 min. The amplified region was purified from 1.5% UltraPura Agarose (Invitrogen, Inc.) gel through QIAquick Gel Extraction Kit (Qiagen, Inc.), and the purified fragments were sequenced using ABI 3730.

### Real-time quantitative RT-PCR

Total RNA of 5 individuals from line 7_2_ and line 6_3_ was extracted from spleen, liver and hypothalamus using RNeasy Midi kit (Qiagen, Inc.). The first strand cDNA was synthesized from total RNA using SuperScript™ III Reverse Transcriptase (Invitrogen). Samples were then analyzed by real time RT-PCR using an iCycler iQ PCR system (Bio-Rad). The real time RT-PCR reactions were performed in a final volume of 20 µl with a QuantiTect SYBR Green PCR Kit (Qiagen) according to the manufacture's instructions. Each reaction has two replicates. The mRNA expression of *DNMT3b* was normalized against the housekeeping gene *GAPDH* (glyceraldehyde 3-phosphate dehydrogenase) cDNA in the corresponding samples.

### Statistical analysis

In this research, point-wise comparison was carried out to analyze the difference of DNA methylation levels at each CpG site between two parental lines and six RCSs. Student's *t* test was used to analyze the difference of RNA expression levels of *DNMTs* between two lines.

In addition, we developed a new method called principal component test to evaluate the significant difference of methylation patterns consisting of continued CpG sites, which was done through the transformation of q-fold principle components (PC) [52]. In brief, the significance test of DNA methylation patterns of the eight populations (lines, tissues and ages of chickens) is conducted on the exact *F* statistic for high-dimension data. The objective is to assess if the two groups to which the n individuals belong are statistically distinguishable.

Let n^(1)^ and n^(2)^, n^(1)^+n^(2)^ = n, represent the numbers of individuals in two populations, respectively, to be compared. Assume each individual has a measure of several continued CpG sites. Denote ***X*** = (***x_1_***,***x_2_***…***x_n_***), a p×n matrix representing methylation percentages on each CpG site.

Assume ***x_i_***∼***N***(***μ_i_***, ***Σ***), the null hypothesis to be tested is
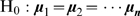
(1)Let us denote 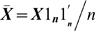, and let ***D*** be a p×q matrix consisting of the first q (1<q<min(n, p)) eigenvectors of the solution of the following general eigenvalue problem
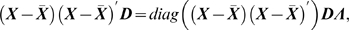
(2)where ***Λ*** is the q×q diagonal matrix of q largest eigenvalues.

If H_0_ holds, the statistic
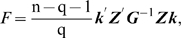
(3)exactly follows F distribution with q and n-q-1 as the degrees of freedom, where ***Z*** = ***D***′***X***, 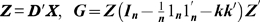. ***k*** is a vector calculated according to following equation
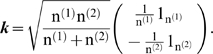
(4)


For a given n and p, the power of this statistic is dependent on the choice of q, the number of principals to be considered. We determined this parameter based on a cumulative energy content (CEC) criterion. That is, the q principals can capture at least 85% variance in the data.

## Supporting Information

Figure S1 mRNA expression levels of DNMT3a and DNMT1 using quantitative RT-PCR. A. The level of DNMT3a in spleen and liver from line 63 at 15 months-old. B. The level of DNMT1 in liver between line 63 and line 72 at 15 months-old. Two replicates for each reaction. n = 5 for each line and tissue.
(0.80 MB DOC)

Figure S2 Predicted transcriptional binding sites at the studied CpG sites region of DNMT3b in the line 63 (A) and line 72 (B). Open boxes show the two CpG (A) to TpG (B) transitions. Underlined sequences are the predicted core regions of transcriptional binding sites using Matinspector software available on website www.genomatix.de. Transcription factors: DMP1, also named DMTF1 (Cyclin D binding myb-like transcription factor 1). In vivo, DMP1 is a physiological regulator of the Arf-p53 pathway, an oncogene-suppressor pathway; PLAG1 (Pleomorphic adenoma gene) encodes a developmentally regulated, SUMOylated and phosphorylated zinc finger transcription factor, recognizes a specific bipartite DNA consensus sequence regulating expression of a spectrum of target genes. PLAG1 is defined by various studies as a tumor-suppressor gene; NUDR, Nuclear DEAF-1-related protein, is a transcriptional regulatory factor with sequence similarity to developmental and oncogenic proteins. NUDR produced a 65–70% repression of the nuclear ribonucleoprotein A2/B1 promoter activity; PAX5, B-cell-specific activator protein. PAX5 is essential for the transcriptional control of B cell commitment, development and function as well as in B cell tumorigenesis.
(1.19 MB TIF)

Figure S3 Exact F test for DNA methylation patterns of DNMT3b. A. P values matrix among two parental lines 63 and 72, as well as six RCSs, C, F, J, L, M and T. n = 5 for each. B. P values matrix among lines and tissues. 63: line 63; 72: line 72; S: spleen; Li: liver. Color bar shows the extent of significance level (P values with −log10(P). e.g., −log10(0.05) = 1.3; −log10(0.01) = 2). Black arrows show P = 0.05.
(1.47 MB TIF)

Figure S4 P values matrix with exact F test for DNA methylation patterns of DNMT3a among lines, tissues and ages. 63: line 63; 72: line 72; S: spleen; Li: liver; Hy: hypothalamus; B: blood cell. 15: 15 months old; 8: 8 weeks old. n = 5 for each. Color bar shows the extent of significance level (P values with −log10(P). e.g., −log10(0.05) = 1.3; −log10(0.01) = 2). Black arrows show P = 0.05.
(1.01 MB TIF)

Figure S5 P values matrix with exact F test for DNA methylation patterns of DNMT1 among lines and tissues. 63: line 63; 72: line 72; S: spleen; Li: liver; Hy: hypothalamus; B: blood cell. n = 5 for each. Color bar shows the extent of significance level (P values with −log10(P). e.g., −log10(0.05) = 1.3; −log10(0.01) = 2). Black arrows show P = 0.05.
(0.95 MB TIF)

Figure S6 BLAT results of chicken DNMT3B (S6A), DNMT3A (S6B) and DNMT1 (S6C) using UCSC Genome Browser. Brown boxes show the first exon that including the analyzed CpG sites in each gene.
(5.39 MB TIF)
