# Supplementary figures and images for: An Integrated Epigenetic and Genetic Analysis of DNA Methyltransferase Genes (DNMTs) in Tumor Resistant and Susceptible Chicken Lines
Source: PLoS One. 2008 Jul 16;3(7):e2672. doi: 10.1371/journal.pone.0002672 (PMC2481300; doi:10.1371/journal.pone.0002672)

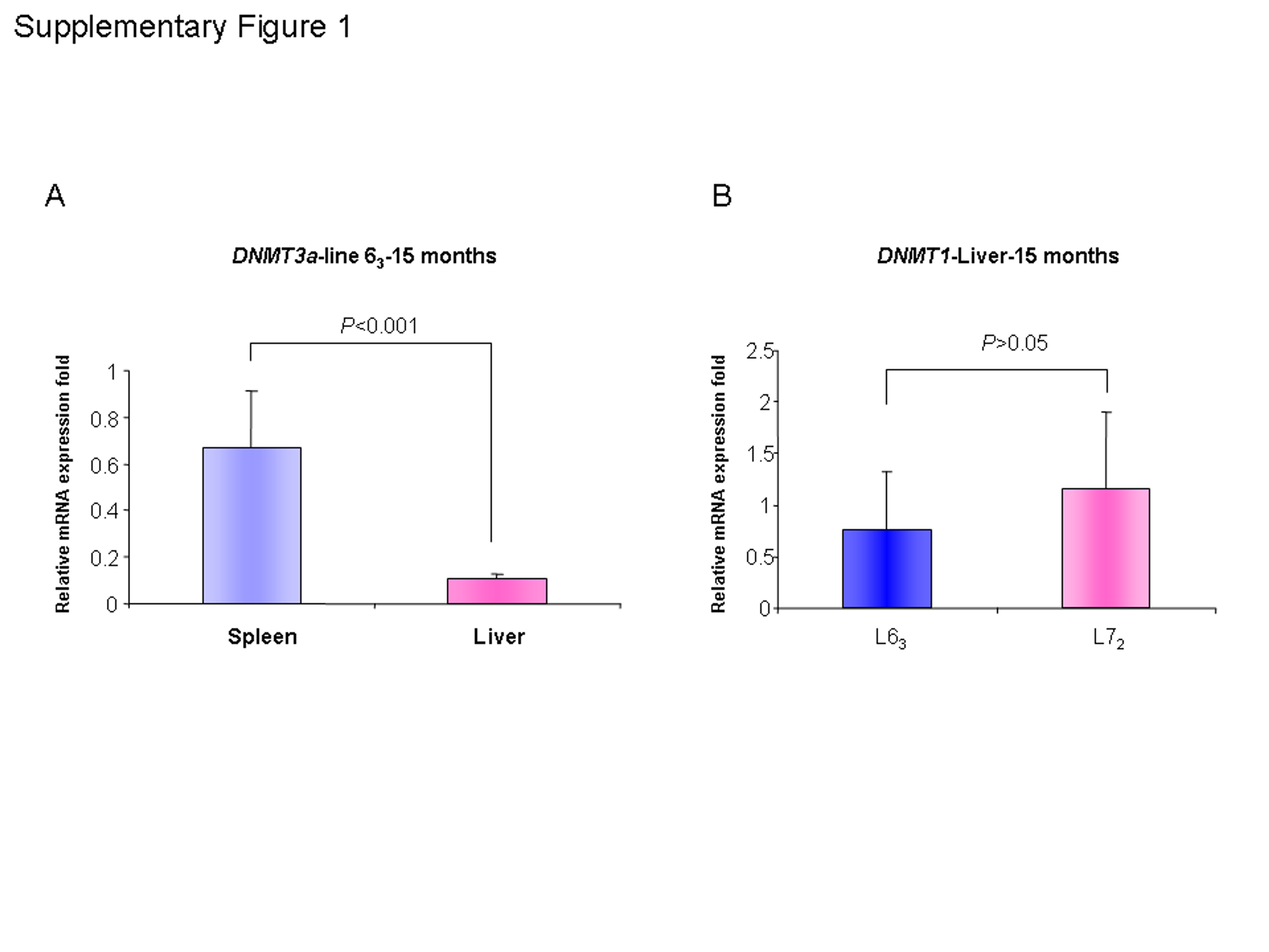

Supplement: Figure S1 — mRNA expression levels of DNMT3a and DNMT1 using quantitative RT-PCR. A. The level of DNMT3a in spleen and liver from line 63 at 15 months-old. B. The level of DNMT1 in liver between line 63 and line 72 at 15 months-old. Two replicates for each reaction. n = 5 for each line and tissue. (0.80 MB DOC) [file pone.0002672.s001.tif]

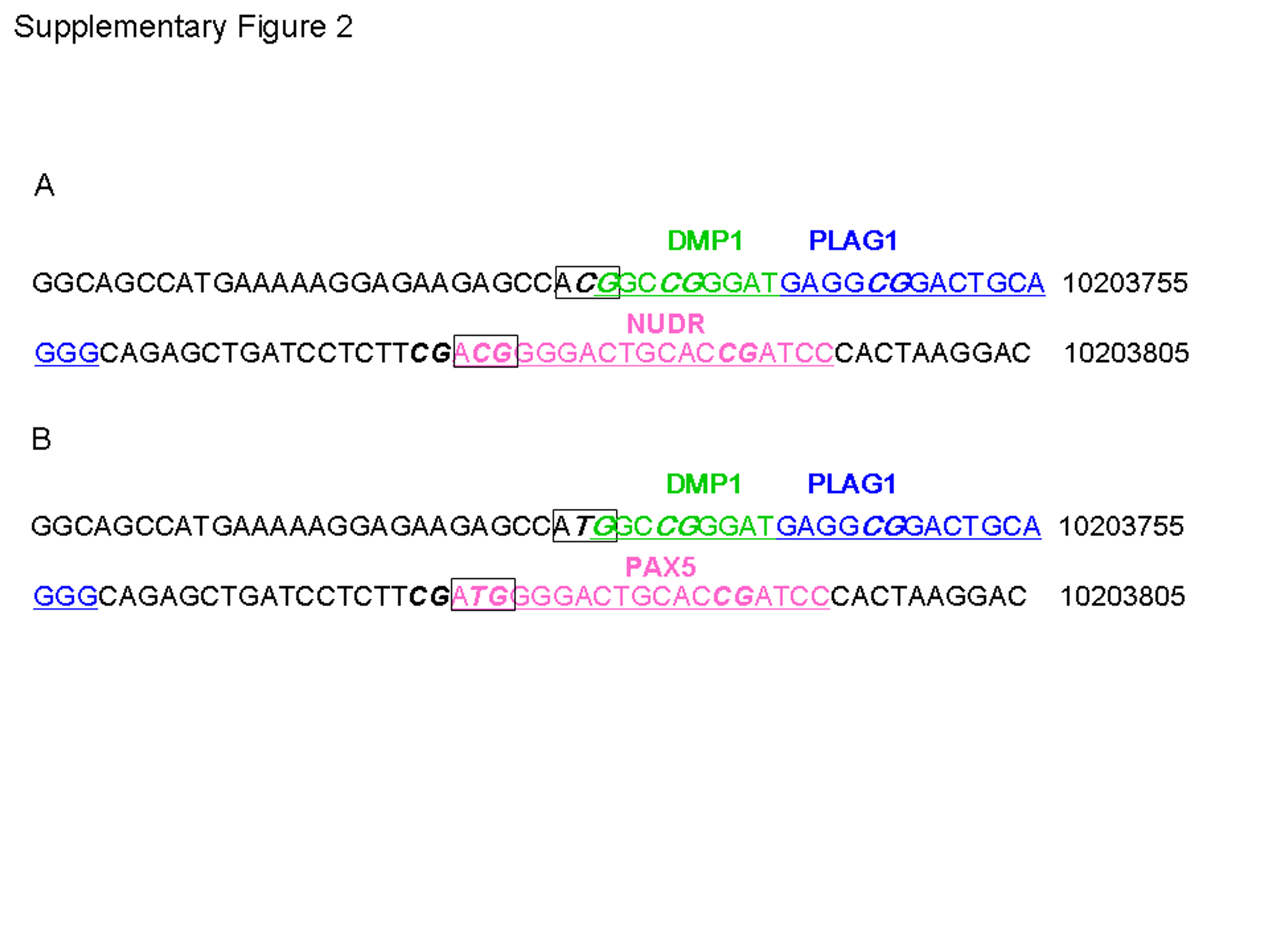

Supplement: Figure S2 — Predicted transcriptional binding sites at the studied CpG sites region of DNMT3b in the line 63 (A) and line 72 (B). Open boxes show the two CpG (A) to TpG (B) transitions. Underlined sequences are the predicted core regions of transcriptional binding sites using Matinspector software available on website www.genomatix.de. Transcription factors: DMP1, also named DMTF1 (Cyclin D binding myb-like transcription factor 1). In vivo, DMP1 is a physiological regulator of the Arf-p53 pathway, an oncogene-suppressor pathway; PLAG1 (Pleomorphic adenoma gene) encodes a developmentally regulated, SUMOylated and phosphorylated zinc finger transcription factor, recognizes a specific bipartite DNA consensus sequence regulating expression of a spectrum of target genes. PLAG1 is defined by various studies as a tumor-suppressor gene; NUDR, Nuclear DEAF-1-related protein, is a transcriptional regulatory factor with sequence similarity to developmental and oncogenic proteins. NUDR produced a 65–70% repression of the nuclear ribonucleoprotein A2/B1 promoter activity; PAX5, B-cell-specific activator protein. PAX5 is essential for the transcriptional control of B cell commitment, development and function as well as in B cell tumorigenesis. (1.19 MB TIF) [file pone.0002672.s002.tif]

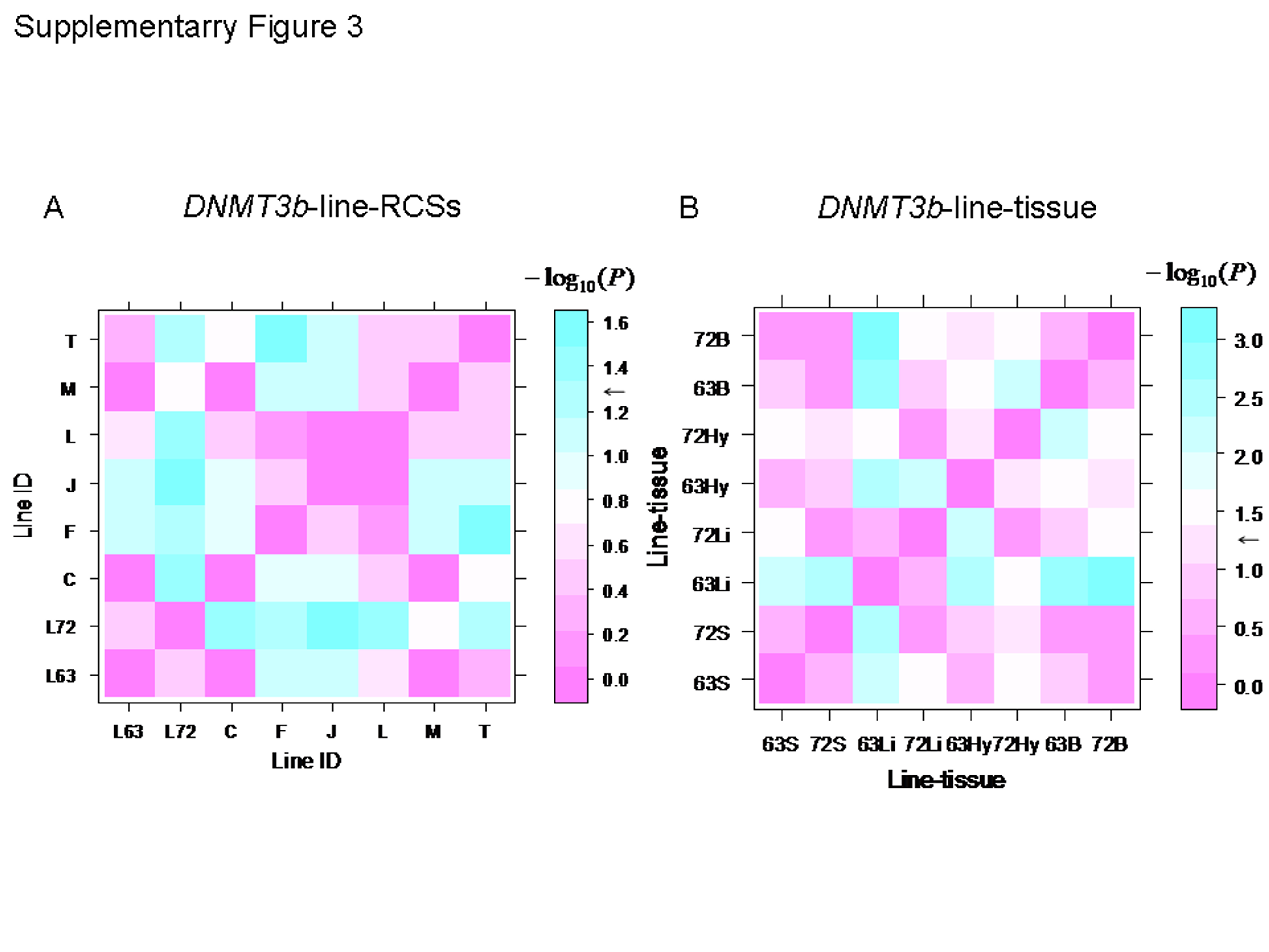

Supplement: Figure S3 — Exact F test for DNA methylation patterns of DNMT3b. A. P values matrix among two parental lines 63 and 72, as well as six RCSs, C, F, J, L, M and T. n = 5 for each. B. P values matrix among lines and tissues. 63: line 63; 72: line 72; S: spleen; Li: liver. Color bar shows the extent of significance level (P values with −log10(P). e.g., −log10(0.05) = 1.3; −log10(0.01) = 2). Black arrows show P = 0.05. (1.47 MB TIF) [file pone.0002672.s003.tif]

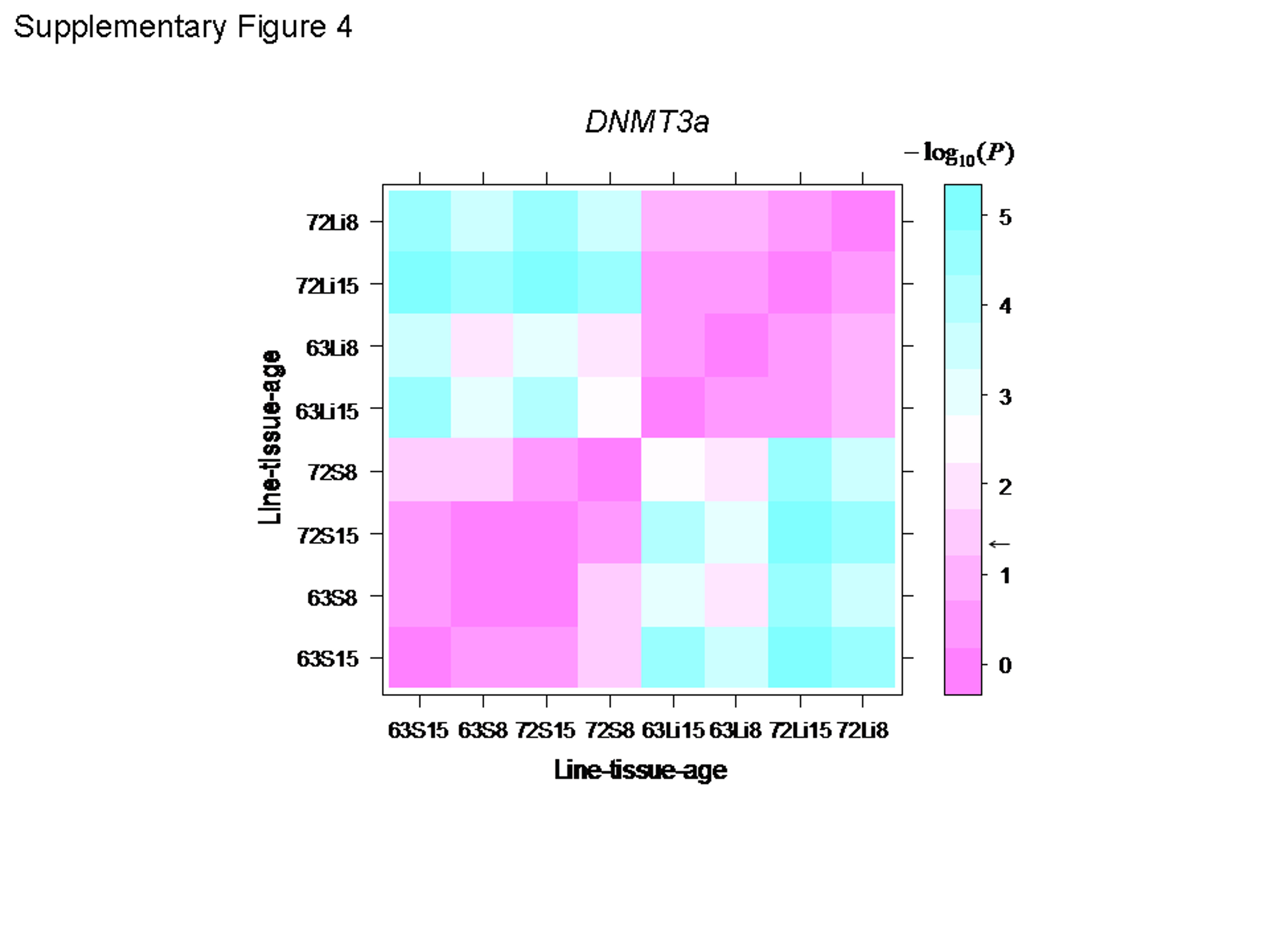

Supplement: Figure S4 — P values matrix with exact F test for DNA methylation patterns of DNMT3a among lines, tissues and ages. 63: line 63; 72: line 72; S: spleen; Li: liver; Hy: hypothalamus; B: blood cell. 15: 15 months old; 8: 8 weeks old. n = 5 for each. Color bar shows the extent of significance level (P values with −log10(P). e.g., −log10(0.05) = 1.3; −log10(0.01) = 2). Black arrows show P = 0.05. (1.01 MB TIF) [file pone.0002672.s004.tif]

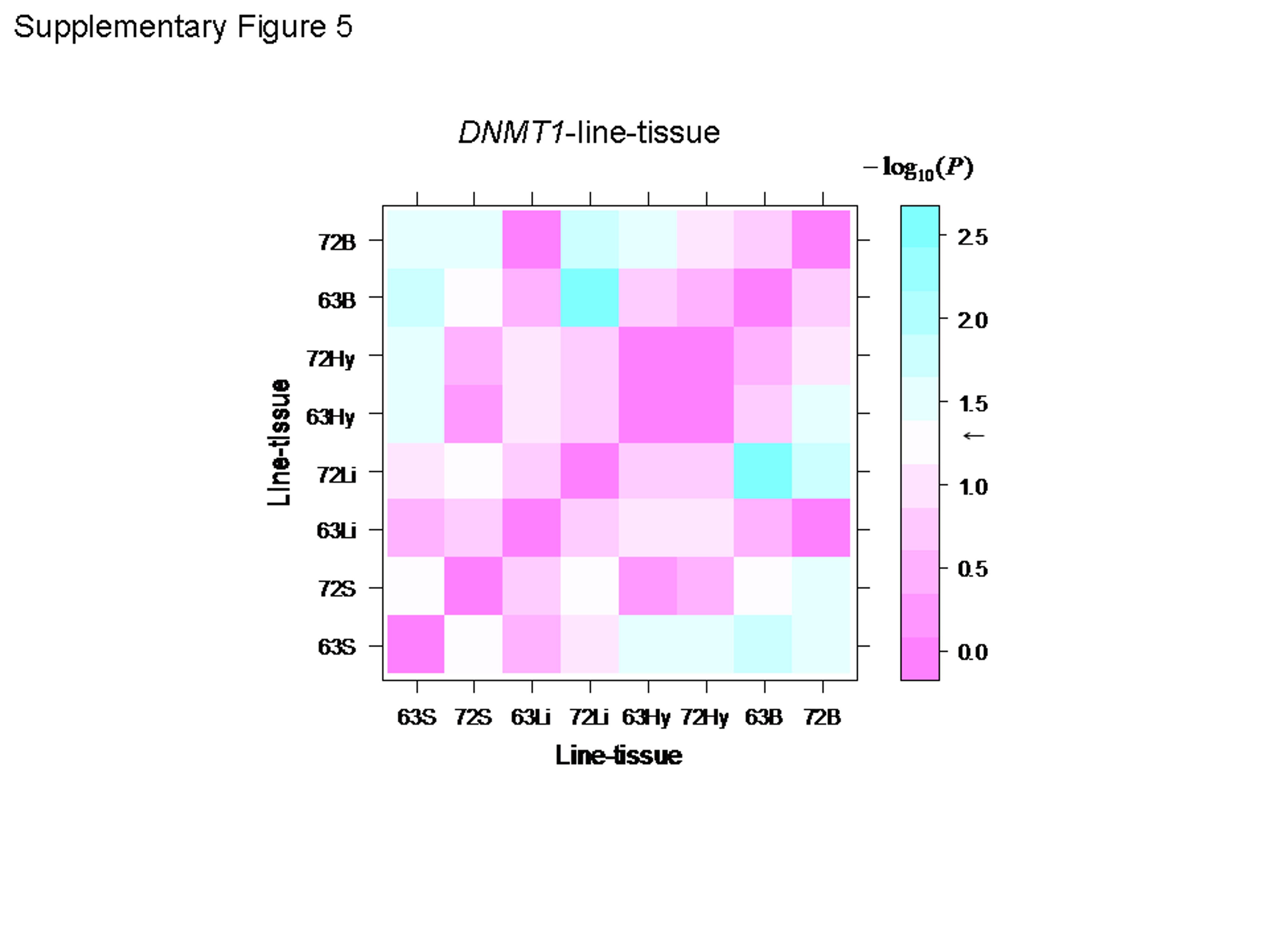

Supplement: Figure S5 — P values matrix with exact F test for DNA methylation patterns of DNMT1 among lines and tissues. 63: line 63; 72: line 72; S: spleen; Li: liver; Hy: hypothalamus; B: blood cell. n = 5 for each. Color bar shows the extent of significance level (P values with −log10(P). e.g., −log10(0.05) = 1.3; −log10(0.01) = 2). Black arrows show P = 0.05. (0.95 MB TIF) [file pone.0002672.s005.tif]

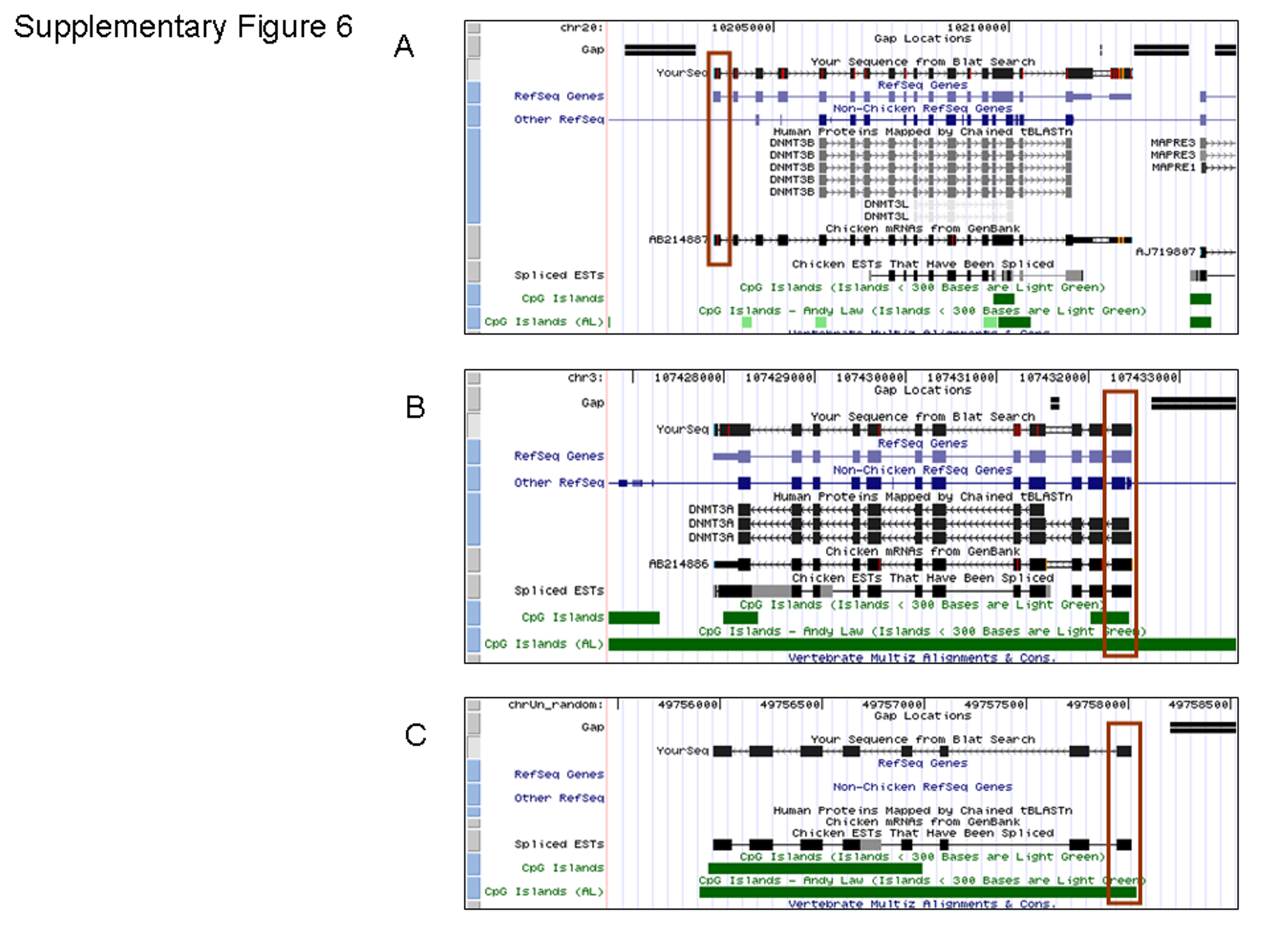

Supplement: Figure S6 — BLAT results of chicken DNMT3B (S6A), DNMT3A (S6B) and DNMT1 (S6C) using UCSC Genome Browser. Brown boxes show the first exon that including the analyzed CpG sites in each gene. (5.39 MB TIF) [file pone.0002672.s006.tif]
